# The wrong drug problem continues

**DOI:** 10.4103/0019-5049.63645

**Published:** 2010

**Authors:** Sarika Katiyar

**Affiliations:** Department of Anesthesiology and Critical Care, Bhopal Memorial Hospital and Research Center, Bhopal - 462 038, India

Sir,

I read with interest the correspondence by Singh *et al*.[1] titled "The wrong drug problem continues". Medication error is a leading cause of morbidity and mortality in hospitalised patients. Due to high potency, variety and frequency of drugs administered to patients undergoing anaesthesia, the potential for errors exists, with disastrous consequences. The major cause of drug error is misidentification of drug ampoules or vials. Confusing, inaccurate or incomplete labels contributed to 21% of the actual or potential drug errors reported to the US Pharmacopoeia practitioners network over a one-year period (1999).[2] The American Society of Anaesthesiologists supports the manufacture and use of pharmaceuticals with labels meeting standards that are consistent with those established by the *American Society for Testing and Materials* (ASTM) International. The main change to the drug label is the introduction of a critical information panel or field. The label presents the generic name of the drug, the total amount per total volume and the drug concentration in black text on a white background. In addition, the drug's proprietary name, manufacturer, lot number, date of manufacture and expiry date should also be included on the label. The text on the label should be designed to enhance the recognition of the drug name and concentration as recommended in the ASTM International standards.[3]

Maximum Contrast between the text and the background should be provided by high contrast colour combinations, as specified in section 6.3.1 of the ASTM International Standards, which also minimise the impact of colour blindness[3] [Table 1]

**Table 1:** Contrasting background for labels

Text	Background
Black	White
Blue	Yellow
White	Blue
Blue	White

Nine classes of drugs commonly used in the practice of anaesthesiology have a standard background colour established by the ASTM International standards for user-applied syringe labels. For these drugs the colour of the container top, label border and any other coloured area on the label, excluding the background, as required for maximum contrast; should be the colour responding to the drug's classification. [3] [Table 2] Essential information including the drugs generic name, concentration and volume of the vial or ampoule should be bar coded at a location on the vial or ampoule, which will not interfere with the labels legibility as specified in the ASTM International Standards.[4]

**Table 2:** Drug class and pantone colours

Drug ClassText	Pantone colour
Induction agents	Yellow
Tranquilizers	Orange
Muscle relaxants	Fluorescent Red
Relaxant antagonistsLung	Florescent Red / White Diagonal stripes
Narcotics	Blue
Narcotic antagonists	Blue / White diagonal stripes
Major tranquilizers	Salmon
Narcotic / Tranquilizer combination	Blue / Salmon
Vasopressors	Violet
Hypotensive agents	Violet / White diagonal stripes
Local anaesthetics	Grey
Anticholinergic agents	Green

As described by Singh *et al*. the drug error was prevented by watching the volume of drug in the syringe. Moreover, the Bupivacaine ampoules (sterile ampoules) packing should not be opened. The packing should be opened only during spinal anaesthesia, as these ampoules are sterile and are to be loaded after all aseptic precautions.

Current evidence indicates that improving clinical communication can also reduce medical error to some extent. Thus, drug errors are an inevitable consequence of the human condition, they occur even among the most conscientious medical professionals.
